# Potential utilization of bagasse as feed material for earthworm *Eisenia fetida* and production of vermicompost

**DOI:** 10.1186/s40064-014-0780-y

**Published:** 2015-01-13

**Authors:** Sartaj Ahmad Bhat, Jaswinder Singh, Adarsh Pal Vig

**Affiliations:** Department of Botanical and Environmental Science, Guru Nanak Dev University, Amritsar, India; Department of Zoology, Khalsa College Amritsar, Punjab, India

**Keywords:** Vermicomposting, Sugar mill waste, Bagasse waste, *Eisenia fetida*, Growth and population

## Abstract

In the present work bagasse (B) i.e waste of the sugar industry, was fed to *Eisenia fetida* with cattle dung (CD) support as feed material at various ratios (waste: CD) of 0:100 (B_0_), 25:75 (B_25_), 50:50 (B_50_), 75:25 (B_75_) and 100:0 (B_100_) on dry weight basis. Co-composting with cattle dung helped to improve their acceptability for *E. fetida* and also improved physico-chemical characteristics. Best appropriate ratio for survival, maximum growth and population buildup of *E. fetida* was determined by observing population buildup, growth rate, biomass, mortality and cocoon formation. Minimum mortality and highest population size of worms was observed in 50:50 (B_50_) ratio. Increasing concentrations of wastes significantly affected the growth and reproduction of worms. Nutrients like nitrogen, phosphorus and sodium increased from pre-vermicompost to post-vermicompost, while organic carbon, and C:N ratio decreased in all the end products of post-vermicomposting. Heavy metals decreased significantly from initial except zinc, iron and manganese which increased significantly. Scanning electron microscopy (SEM) was used to recognize the changes in texture in the pre and post-vermicomposted samples. The post-vermicomposted ratios in the presence of earthworms validate more surface changes that prove to be good manure. The results observed from the present study indicated that the earthworm *E. fetida* was able to change bagasse waste into nutrient-rich manure and thus play a major role in industrial waste management.

## Introduction

In Indian economy the sugar mill has an essential place and contributes considerably to its exports earnings. India is the second largest producer of sugar and its by-products amongst the 83 sugarcane producing countries in the world (Rao 2005). The industry succeed a very impressive gain as it has 1062 sugar industries of large to medium size as compared to 138 during 1950–1951 (Sangwan et al. 2008). About 270 million tons of sugar cane per year is produced in India (Zeyer et al. 2004). During the manfacturing process large amount of by-products such as bagasse, pressmud and sugar cane residue are produced. Bagasse (B) is the fibrous waste produced in the sugarcane juice extraction process. It constitutes cellulose (50%), hemicelluloses (25%) and lignin (25%) (Ezhumalai and Thangavelu 2010). Bagasse is a highly homogeneous material constitute around 30-40% of pith fibre, which is obtained from the core of the plant. The estimated generation is 0.25-0.30 ton per ton of sugarcane (Pessoa et al. 1997). In agro-residue based pulp and paper mills, bagasse is used as a raw material. Disposal of bagasse by dumping is unattractive process because of large requirement of land and pollution concerns. Vermicomposting is the good method of converting organic wastes into environmentally friendly products. It is a bio-oxidative process entails the combined action of earthworms and microbes. Earthworms ingest, break and digest waste and converts into finer, humified, microbially active material by the activity of earthworms and microbes (Khwairakpam and Bhargava 2009). The final product i.e. vermicompost is a granulated material with high porosity and water holding capacity.

In the present work, bagasse of sugar industry was subjected to vermicomposting for its bioremediation. Co-composting with cattle dung helped to improve their acceptability for *Eisenia fetida* and also improved physico-chemical characteristics. Growth and fecundity of *E. fetida* were taken as parameters of appropriate feed ratios. Efficiency of *E. fetida* for recuperate nutrients was analysed by physico-chemical parameters in the waste and vermicompost after bioconversion. Scanning electron microscopy (SEM) was applied to recognize the changes in surface morphology in the pre and post-vermicomposted samples.

## Materials and methods

### Bagasse, Cattle dung and *Eisenia fetida*

Fresh B was obtained from Rana Sugars Limited, Amritsar, Punjab, India. CD was obtained from a dairy farm situated in the vicinity of the university. Young non-clitellated *E. fetida* with an average weight 0.05 g were randomly picked from a stock culture maintained in the vermicomposting unit of the Department of Botanical and Environmental Sciences, Guru Nanak Dev University, Amritsar, Punjab, India. The initial physico-chemical parameters of B and CD are given in Table 1.

**Table 1:** **Initial physico-chemical properties of bagasse and cattle dung**

**Physico-chemical parameters**	**Bagasse**	**Cattle dung**
pH	6.55 ± 0.07	8.35 ± 0.08
EC (mS/cm)	1.1 ± 0.1	4.13 ± 0.17
TKN (%)	0.26 ± 0.01	1.34 ± 0.01
TOC (%)	55.53 ± 0.27	46.28 ± 0.52
C:N ratio	213.57 ± 10.6	34.53 ± 0.26
TAP (%)	0.20 ± 0.05	0.59 ± 0.06
TK (%)	3.19 ± 0.04	2.23 ± 0.04
TNa (%)	1.08 ± 0.10	8.09 ± 0.28
Zn^a^	21.54 ± 0.24	63.52 ± 1.62
Cu^a^	18.6 ± 1.41	55.57 ± 3.73
Cr^a^	26.03 ± 2.64	56.77 ± 4.28
Fe^a^	249.5 ± 7.67	1483 ± 6.44
Mn^a^	16.79 ± 1.72	83.09 ± 0.38

^a^Weight in mg/Kg.

### Experimental setup

B and CD were mixed and subjected to vermicomposting in the concentrations of 0:100 (B_0_), 25:75 (B_25_), 50:50 (B_50_), 75:25 (B_75_) and 100:0 (B_100_) (waste: CD) on a dry weight basis (Table 2). The experiments were run in plastic trays (28 × 3 × 6 cm) in triplicates under the vermicomposting unit of the university. The total weight of each tray was kept at 2 kg. The trays were covered with hessian cloth and mixtures were turned over manually every 24 hours for 14 days in order to remove volatile toxic gases. After 14 days, 50 young non-clitellated *E. fetida* were added to the feed mixtures. The moisture content was maintained to 60-70% throughout the experiment by watered regularly. Earthworms, cocoons and hatchlings were sorted and counted manually at the interval of 15 days and then placed back in the trays after sampling. At the end of the experiment, worms, cocoons and hatchlings were taken out and put back in separate stock culture. The vermicompost was air dried, sieved and stored at low temperature i.e 10°C for physico-chemical analysis.

**Table 2:** **Percentages of bagasse and cattle dung in different proportions on dry weight basis**

**Feed mixtures**	**Bagasse (B)**	**Cattle dung (CD)**
B_0_	0	100
B_25_	25	75
B_50_	50	50
B_75_	75	25
B_100_	100	0

### Physico-chemical analysis

pH and electrical conductivity (EC) of initial feed mixture and final products were measured in distilled water suspension of each concentration in the ratio of 1:10 (W/V) using Systronics μ pH system 362 and Systronics conductivity meter-304, respectively. Total organic carbon (TOC) was determined after burning the 0.5 g of waste in a muffle furnace at 550°c for 60 min as described by Nelson and Sommers (1996). Micro-Kjeldhal method of AOAC (2000) was used for measuring nitrogen after digesting the waste in digestion mixture (H_2_SO_4_ + K_2_SO_4_:CuSO_4_:SeO_2_ in 10:4:1). The method described by John (1970) was used for measuring total available phosphorus (TAP) using Systronics double beam spectrophotometer 2202, total potassium (TK) and sodium (TNa) was measured by using a Systronics flame photometer-128 after digesting the samples in diacid mixture (HClO_4_:HNO_3_ in 4:1 ratio). Heavy metals were measured by Agilent 240 FS AA model Atomic Absorption Spectrophotometer in the digested samples.

### Scanning electron microscopy

The pre and post-vermicomposted samples were analyzed to study the texture using Zeiss EVO LS-10 electron microscope. About 2–3 mg sample of particle size 300 mm was spread uniformly over the stub with the help of a double sided adhesive tape and subsequently coated with gold using sputter coater and imaged under SEM at different magnifications.

### Statistical analysis

The differences among various feed mixtures were calculated by One-way ANOVA followed by Tukey’s HSD test. Student’s paired *t*-test was used to assess differences between pre and post-vermicompost values of various physico-chemical parameters. Experiment was run in triplicate and statistical analysis was done on triplicate values. Statistical analysis was done with the help of SPSS version 16.0 and Minitab version 14.0 computer software programs.

## Results and discussion

### Increase in number and biomass of earthworm

**Figure 1:**
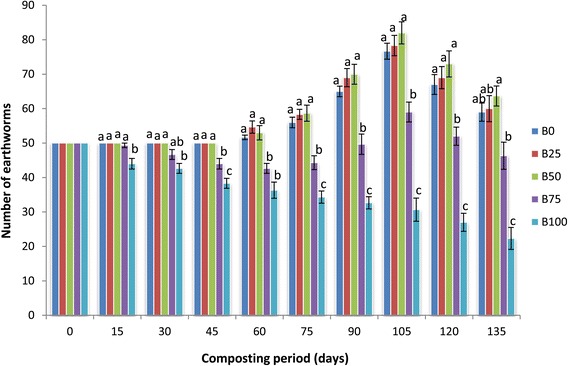
**Mean values of number of earthworms followed by different letters in a same day are significantly different (one-way ANOVA; Tukey’s test, p ≤ 0.05) in different feed mixtures of bagasse and cattle dung.**

**Figure 2:**
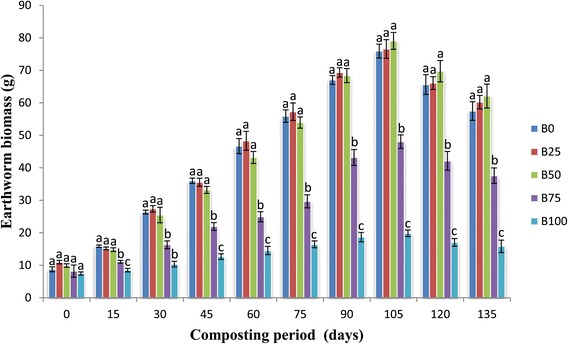
**Mean values of earthworm biomass (g) followed by different letters in a same day are significantly different (one-way ANOVA; Tukey’s test, p ≤ 0.05) in different feed mixtures of bagasse and cattle dung.**

Population buildup in various mixtures of bagasse was significantly different (p <0.05). Earthworm number started increasing on 60th day in B_0_, B_25_ and B_50_ mixtures and the increase continued till the 105th day of experiment. At the 105th day of experiment maximum increase was observed in B_50_ (82 ± 3.21) followed by B_25_ (78.33 ± 2.96) and B_0_ mixture (76.67 ± 2.33) (Figure 1). The earthworm number decreased from 15th day of experiment in B_100_ mixture. In B_75_ mixture, however, a decline in numbers up to 60th day was followed by an increase up to 105th day. Only 46.33 ± 3.93 and 22.33 ± 3.18 worms were present on the 135th day in B_75_ and B_100_ feed mixtures. The maximum earthworm biomass was in the B_50_ (79.04 ± 2.60) feed mixture on the 105th day of experiment and minimum in the B_100_ (15.83 ± 1.93) feed mixture on the 135th day of experiment (Figure 2). Survival, biomass formation and reproduction of earthworms are the best sign to analyse the vermicomposting process. In the present study the number of worms in bagasse started to decrease between 105th and 135th days of experiment. At this time the vermicompost started granulating on the surface which demonstrates finishing of food in the mixtures. Continuous decline of earthworms was observed in the higher mixtures of bagasse (B_100_) till the end of the experiment, which hints towards its toxicity for earthworms even after 135 days. The survival, growth rate and reproduction potential of earthworms has been affected by the type, palatability and quality of food (Tripathi and Bhardwaj 2004; Gajalakshmi et al. 2005). Our results corroborate with the findings of Bhat et al. (2014) that an increasing content of pressmud in the feed mixture brought a decrease in the number of earthworms. Ideal ratio of bagasse with cattle dung was 50: 50, as final vermicompost started granulating on its surface earliest (90–100 days) and this ratio was also found to be suitable for growth and population buildup of *E. fetida*. In the present study also worm biomass increased in all feed mixtures of bagasse. Rathinamala et al. (2008) also reported increase in body weight of *Eisenia eugeniae* feeding on different organic substrates. Presence of fungi during vermicomposting processes becomes additional food to the worms which contributes to the higher weight of the worms (Pramanik and Chung 2011).

### Cocoon production

**Figure 3:**
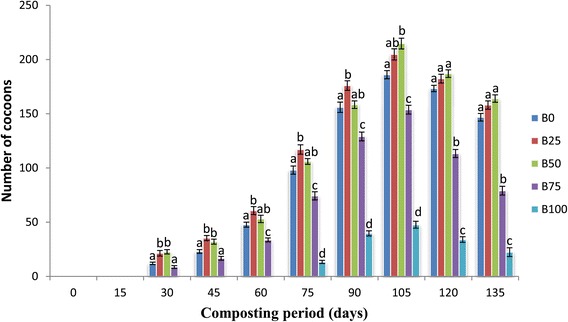
**Mean values of number of cocoons followed by different letters in a same day are significantly different (one-way ANOVA; Tukey’s test, p ≤ 0.05) in different feed mixtures of bagasse and cattle dung.**

The number of cocoons in different mixtures of bagasse was significantly different (p <0.05). Cocoon formation started after 30th day in B_0_, B_25,_ B_50_ and B_75_ and after 75th day in B_100_. The maximum number of cocoons were observed in B_50_ (214.7 ± 4.91) on 105th day and minimum in B_100_ (22.33 ± 4.05) on 135th day (Figure 3). Quality of the feed mixture determines the growth of earthworms and onset as well as the rate of cocoon formation. Cocoon production was relatively less in higher concentrations (B_75,_ B_100_) as these mixtures contain less nitrogen content as contrast to the other feed mixtures, thus it could be reason for less cocoon production in this mixture. The results are also supported by the findings of Suthar (2007) that the nitrogen amount of the substrates as an important factor related to cocoon production. Fayolle et al. (1997) have also observed that food source play a crucial role on cocoon formation. Higher concentrations declined rate of degradation and drastically affected the earthworms as it delayed as well as decreased cocoon formation. Chauhan and Singh (2012) have also reported that the various binary combination of buffalo dung with agro-wastes caused a significant growth of *E. fetida* and increase in cocoons production.

### Hatchling formation and weight of hatchlings

**Figure 4:**
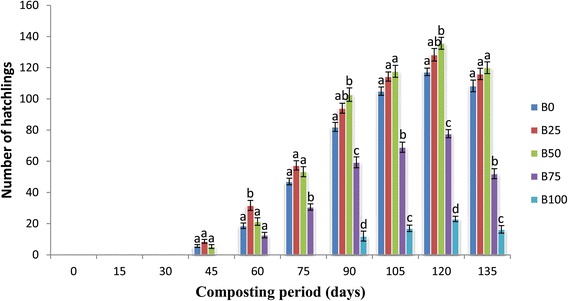
**Mean values of number of hatchlings followed by different letters in a same day are significantly different (one-way ANOVA; Tukey’s test, p ≤ 0.05) in different feed mixtures of bagasse and cattle dung.**

**Figure 5:**
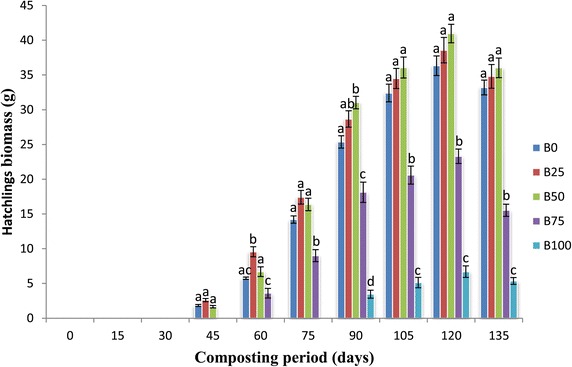
**Mean values of hatchlings biomass (g) followed by different letters in a same day are significantly different (one-way ANOVA; Tukey’s test, p ≤ 0.05) in different feed mixtures of bagasse and cattle dung.**

Hatchling formation was significantly different (p <0.05). Hatchlings were observed for the first time on 45th day in B_0,_ B_25_ and B_50_, on 60th day in B_75_ and on 90th day in B_100_ mixture. The maximum number of hatchlings were observed in B_50_ (135.7 ± 3.84) on 120th day and minimum in B_100_ (16.33 ± 2.40) on 135th day of experiment (Figure 4). Maximum hatchling biomass was observed in B_50_ (40.97 ± 1.33) on the 120th day of experiment and minimum in the B_100_ (5.36 ± 0.49) feed mixture (Figure 5). Number of hatchlings was lesser in feed mixtures B_75_ and B_100_ as compared to lower proportions due to low production of cocoons. Increase in hatchling formation during the present study gets supported by Kaur et al. (2010) and Chauhan and Singh (2013).

### Physico-chemical characteristics of the mixtures

The physico-chemical analysis also showed significant difference (p <0.01) with varying ratio of waste in different concentrations (Table 3). In the present study decrease in pH was observed. The maximum decrease in pH was in B_0_ (14.73%) and minimum (6.04%) in B_75_ feed mixture. In B_100_ mixture, however an increase in pH (8.85%) was observed. The decline in pH during vermicomposting is due to the mineralization of nitrogen and phosphorus compounds and the production of humic and fulvic acids (Ndegwa and Thompson 2000). The decrease in pH by the earthworm in the feed mixtures (B_0,_ B_25,_ B_50_) may be due to the earthworm related gut microorganisms responsible for the production of organic acids. Increase in EC was significantly different (p < 0.01). The maximum increase in EC was in B_100_ (102.02%) and least in B_25_ (19.17%). The increase in EC for the mixtures of bagasse was B_100_ > B_75_ > B_50_ > B_0_ > B_25_. EC was increased in all the concentrations of bagasse. Karmegam and Daniel (2009) also reported an increase in EC during vermicomposting and is due to the increase in the soluble salt level resulting from the mineralization action of worms and microbes present in the gut of earthworms and those in the organic substance. Decreased in organic carbon in the feed ratios seems to be responsible for the increase in EC of the mixtures. Increase in Total Kjeldhal Nitrogen (TKN) was increased significantly in the products of bagasse (p <0.01). The maximum increase in TKN was in B_100_ (230.76%) and minimum (49.25%) in B_0_ mixture. Per cent increase in nitrogen of bagasse was in the order of B_100_ > B_75_ > B_50_ > B_25_ > B_0_. In the present study, TKN increased from initial in all the mixtures of bagasse. Cynthia and Rajeshkumar (2012) also reported an increase in TKN of sugar mill effluent and is due to the decomposition of waste by worms to accelerate the nitrogen mineralization process. Earthworm enriches the nitrogen content of vermicompost through decaying tissues of dead earthworms and microbial mediated nitrogen transformation in vermicomposting systems results in further increase of nitrogen (Suthar 2007). TOC of bagasse was decreased significantly from initial in all the feed ratios (p <0.01). The maximum percentage decrease was in B_50_ (35.50%) and least in B_100_ mixture (10.80%). The trend of decline in TOC of bagasse was in the order of B_50_ > B_25_ > B_0_ > B_75_ > B_100_. The ingested feed mixtures would be break down and homogenized by *E. fetida* through muscular activity leading to an increase in surface area for microbial action leading to the decline of TOC content (Suthar and Singh 2008). Prakash and Karmegam (2010) and Bhat et al. (2014) have also observed decrease in TOC during vermicomposting of sugar mill sludge. C:N ratio was found to decrease significantly in all the feed ratios of bagasse (p <0.01). Maximum decrease in C: N ratio was observed in B_50_ mixture (73.35%), whereas, it was minimum in B_0_ mixture (51.08%). Per cent decline in C: N ratio of bagasse was in the order of B_50_ > B_100_ > B_25_ > B_75_ > B_0_. The C:N ratio shows the waste stabilization and mineralization during the process of vermicomposting. Decline in C:N ratio was due to higher loss of carbon through microbial respiration in the form of CO_2_ along with an increase in nitrogen and stabilization of waste by the action of worms (Hait and Tare 2011; Vig et al. 2011). Similar results were also observed by Bhat et al. (2013, 2014). TAP was increased significantly in all the feed ratios of bagasse (p <0.01). Per cent increase over initial was maximum in B_50_ mixture (88.88%), whereas, it was minimum in B_75_ mixture (42.85%). Per cent increase in TAP of bagasse was in the order of B_50_ > B_0_ > B_100_ > B_25_ > B_75_. The increased phosphorus content in vermicompost clearly indicates earthworm mediated phosphorus mineralization (Suthar 2009). Bayon and Binet (2006), observed that an increase in rise of phosphate content of vermicompost was due to presence of alkaline phosphates in the worm casts. TK increased significantly (p <0.01), except for B_0_. The increase was maximum in B_50_ mixture (12.09%), and minimum in B_75_ mixture (1.40%). Increase in the TK of bagasse was in the order of B_50_ > B_25_ > B_100_ > B_75_ mixtures. Variation in TK concentration among the mixtures has been related to differences in the chemical nature of the initial waste. Increase in TK in bagasse correlates with findings of Adi and Noor (2009) that the solubilization of insoluble potassium is due to the formation of acid during waste decomposition by microbes. Suthar (2008) and Yadav et al. (2010) also observed that vermicomposting of wastes significantly increased the potassium concentration. TNa was significantly increased (p <0.01) in the final product of Vermicomposting. Per cent increase over initial was maximum in B_100_ mixture (134.25%), and minimum in B_25_ mixture (19.52%). The increase in TNa of the feed mixtures was in the order of B_100_ > B_75_ > B_0_ > B_50_ > B_25_. Increased in TNa in the present study gets supported by Singh et al. (2010, 2014) and Vig et al. (2011). There was a significant decrease (p <0.05) in heavy metals over initial except Zn, Fe and Mn. Maximum decline of heavy metals was 25.62% and 16.69% for Cu, 62.88% and 8.62% for Cr. Zn, Fe and Mn increased significantly in all the feed ratios of bagasse. Maximum increase was 185.33% for Zn, 84.0% for Fe, 51.24% for Mn. Heavy metals decreased from initial except some elements. Heavy metal reduction accompany with an increase in the weight of worms. Body tissues of earthworms especially chloragocytes and the intestinal microflora have the capacity to detoxity most of the heavy metals and in the present study reduction may be due to the accumulation of these heavy metals by the body tissues of worms. Metabolic conversion of highly toxic forms of heavy metals to nontoxic form has been observed in *E. fetida* (Arillo and Melodia 1991; Fischer and Koszorus 1992).

**Table 3:** **Initial and final nutrient content (mean ± S.E.) and percent change over initial of different proportions of bagasse and cattle dung**

**Feed mixtures of bagasse**						
**Nutrient**		**B**_**0**_	**B**_**25**_	**B**_**50**_	**B**_**75**_	**B**_**100**_
**pH**	Initial	8.35 ± 0.08	8.25 ± 0.02	8.13 ± 0.04	7.77 ± 0.04	6.55 ± 0.07
	Final	7.12 ± 0.05*	7.08 ± 0.08**	7.41 ± 0.04*	7.30 ± 0.04*	7.13 ± 0.06
	% change	−14.73	−14.18	−8.85	−6.04	8.85
**EC (mS/cm)**	Initial	4.13 ± 0.17	3.86 ± 0.08	3.23 ± 0.14	1.63 ± 0.08	1.1 ± 0.1
	Final	5.46 ± 0.14*	4.60 ± 0.11**	4.90 ± 0.11**	2.96 ± 0.12*	2.23 ± 0.12*
	% change	32.20	19.17	51.70	81.59	102.72
**TKN** ^**a**^	Initial	1.34 ± 0.01	0.83 ± 0.02	0.69 ± 0.05	0.43 ± 0.01	0.26 ± 0.01
	Final	2.0 ± 0.03**	1.77 ± 0.06**	1.67 ± 0.04**	1.06 ± 0.03**	0.86 ± 0.04**
	% change	49.25	113.25	142.02	146.51	230.76
**TOC** ^**a**^	Initial	46.28 ± 0.52	49.92 ± 0.07	50.30 ± 0.10	54.25 ± 0.36	55.53 ± 0.27
	Final	33.79 ± 0.36**	32.25 ± 0.65**	32.44 ± 0.56**	44.51 ± 0.27**	49.53 ± 0.35*
	% change	−26.98	−35.39	−35.50	−17.95	−10.80
**C/N ratio**	Initial	34.53 ± 0.26	60.14 ± 2.39	72.89 ± 5.52	126.16 ± 4.05	213.57 ± 10.6
	Final	16.89 ± 0.43**	18.22 ± 0.78**	19.42 ± 0.62*	41.99 ± 1.28**	57.59 ± 2.86**
	% change	−51.08	−69.70	−73.35	−66.71	−73.03
**TAP** ^**a**^	Initial	0.59 ± 0.06	0.48 ± 0.03	0.36 ± 0.03	0.28 ± 0.04	0.20 ± 0.05
	Final	1.08 ± 0.05**	0.71 ± 0.05	0.68 ± 0.04**	0.40 ± 0.03	0.30 ± 0.03
	% change	83.05	47.91	88.88	42.85	50.0
**TK** ^**a**^	Initial	2.23 ± 0.04	2.38 ± 0.03	2.48 ± 0.07	2.85 ± 0.02	3.19 ± 0.04
	Final	1.96 ± 0.06	2.47 ± 0.04	2.78 ± 0.04	2.98 ± 0.04*	3.26 ± 0.06
	% change	−12.10	3.78	12.09	1.40	2.19
**TNa** ^**a**^	Initial	8.09 ± 0.28	5.84 ± 0.15	5.09 ± 0.21	2.30 ± 0.09	1.08 ± 0.10
	Final	13.42 ± 0.21**	6.98 ± 0.27*	7.75 ± 0.13*	3.88 ± 0.07**	2.53 ± 0.14*
	% change	65.88	19.52	52.25	68.69	134.25
**Zn** ^**b**^	Initial	63.52 ± 1.62	45.63 ± 0.55	34.8 ± 1.42	32.68 ± 0.59	21.54 ± 0.24
	Final	120.6 ± 1.28**	130.2 ± 3.31**	115.5 ± 3.06**	84.09 ± 6.19*	60.63 ± 3.13**
	% change	89.86	185.33	231.89	157.31	181.47
**Cu** ^**b**^	Initial	55.57 ± 3.73	37.47 ± 1.31	32.17 ± 1.45	25.93 ± 1.59	18.6 ± 1.41
	Final	41.33 ± 0.92*	29.57 ± 1.07	25.4 ± 2.17	21.6 ± 1.41	15.47 ± 1.32
	% change	−25.62	−21.08	−21.04	−16.69	−16.82
**Cr** ^**b**^	Initial	56.77 ± 4.28	41.47 ± 1.45	18.5 ± 1.07	13.1 ± 1.38	26.03 ± 2.64
	Final	21.07 ± 1.33**	26.83 ± 1.29**	15.43 ± 0.95	11.97 ± 0.61	22.77 ± 1.16
	% change	−62.88	−35.30	−16.59	−8.62	−12.52
**Fe** ^**b**^	Initial	1483 ± 6.44	760.1 ± 10.98	698.8 ± 6.80	334 ± 6.15	249.5 ± 7.67
	Final	899 ± 11.15**	998.9 ± 11.94**	1030 ± 14.44**	542.5 ± 6.06**	459.1 ± 7.28**
	% change	−39.37	31.41	47.39	62.42	84.0
**Mn** ^**b**^	Initial	83.09 ± 0.38	54.13 ± 0.71	45.34 ± 2.98	24.6 ± 2.14	16.79 ± 1.72
	Final	181.4 ± 1.44**	81.87 ± 3.67*	66.7 ± 1.94*	35.1 ± 2.80	20.67 ± 2.34
	% change	118.31	51.24	47.11	42.68	23.10

^a^Concentrations in %.

^b^Weight in mg/kg.

Significance level was determined by student’s *t*-test. *p ≤ 0.05. **p ≤ 0.01.

### Scanning electron microscopy

**Figure 6:**
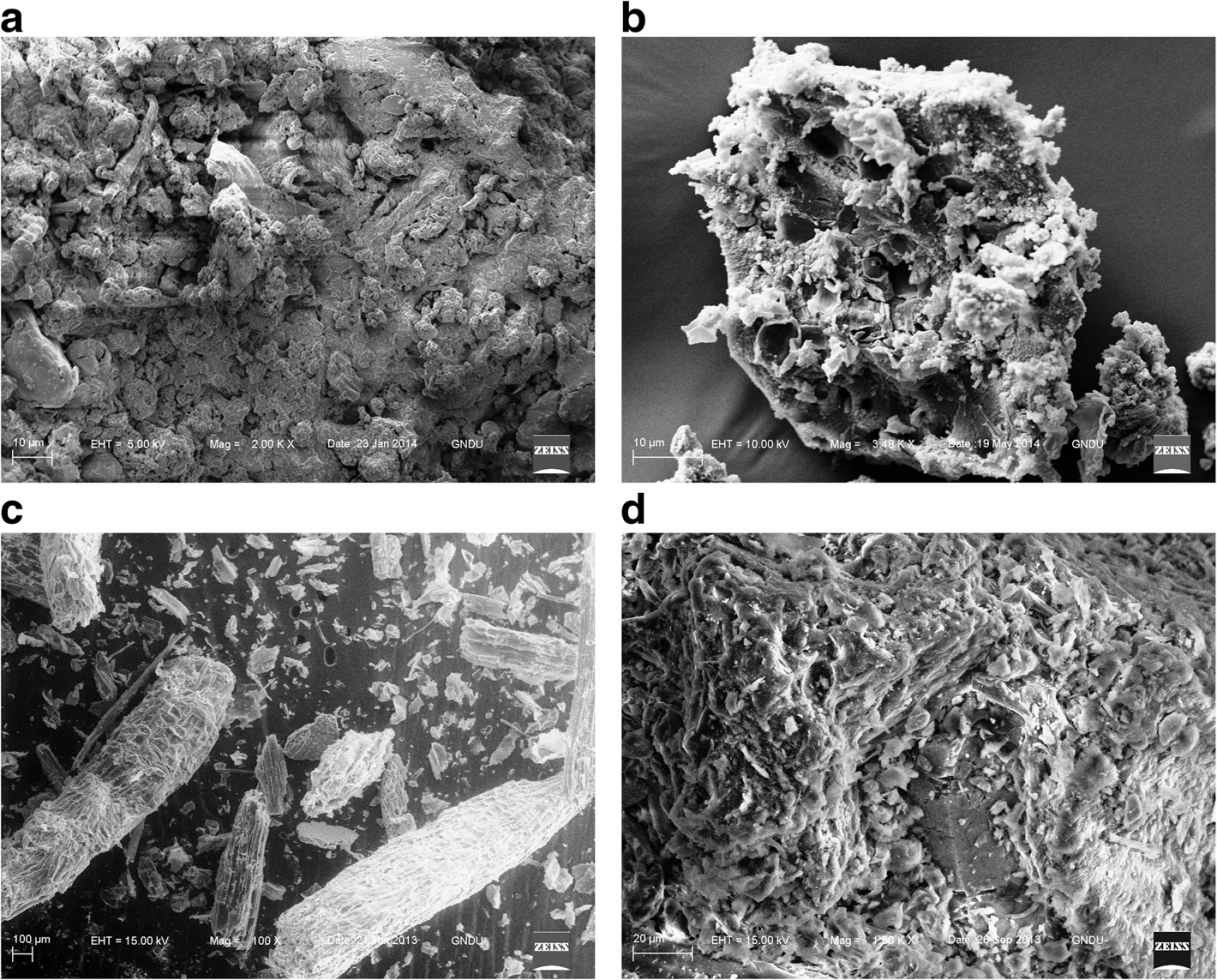
**Scanning Electron Microscopy. a)** Pre-vermicomposted cattle dung. **b)** Post-vermicomposted cattle dung. **c)** Pre-vermicomposted bagasse. **d)** Post-vermicomposted bagasse.

SEM was applied to characterize the pre and post-vermicomposted sample of 100% cattle dung (Figure 6a, b) and pre and post-vermicomposted bagasse (Figure 6c, d) to recognize the changes in texture. In the pre-vermicomposted samples the aggregates of biomass were arranged into cellulose fibres and the protein matrix was strongly bound. However, in the post-vermicomposted samples the protein and lignin was disaggregated by earthworms. Earthworms break the waste in the gut that contains various microbes which helps in progressive degradation. The post-vermicomposted mixtures confirmed the presence of greater numbers of surface irregularities, which indicated that the vermicomposting approach resulted in good vermicompost manure with high porosity and nutrient availability.

## Conclusion

Vermicomposting of bagasse i.e waste of the sugar industry was carried out using earthworm *E. fetida*. Nutrients like TKN, EC, TAP, TNa increased from initial whereas there was decrease in TOC, pH, C:N ratio, and heavy metals. The best growth and reproduction of *E. fetida* were observed in 50:50 (B_50_) mixture. However, greater concentrations of bagasse waste significantly affected the growth and reproduction of *E. fetida*. SEM results also validate the maturity of compost. Vermicomposting could be introduced as efficient technology to convert bagasse waste into nutrient rice manure.
